# Epidemiological Assessment of Depression, Activities of Daily Living and Associated Factors in Elderly Individuals Aged 65 Years and Older: Evidence from a Population-Based Study

**DOI:** 10.3390/jcm14082853

**Published:** 2025-04-21

**Authors:** Mehmet Emin Arayici, Ali Kose, Suleyman Dolu, Sema Gultekin Arayici, Gizem Gedik, Beyza Nur Kilic, Ozum Erkin

**Affiliations:** 1 Department of Public Health, Faculty of Medicine, Dokuz Eylül University, Inciralti-Balcova, Izmir 35340, Turkey; 2 Department of Biostatistics and Medical Informatics, Faculty of Medicine, Dokuz Eylül University, Inciralti-Balcova, Izmir 35340, Turkey; 3 Department of Public Health, Institute of Health Sciences, Dokuz Eylül University, Inciralti-Balcova, Izmir 35340, Turkey; 4 Department of Internal Medicine, Faculty of Medicine, Dokuz Eylül University, Inciralti-Balcova, Izmir 35340, Turkey; 5 Department of Clinical Psychology, Institute of Social Sciences, Ege University, Bornova, Izmir 35040, Turkey; 6 Department of Psychology, Faculty of Arts and Sciences, Atilim University, Incek, Ankara 06830, Turkey; 7 Department of Public Health Nursing, Faculty of Health Sciences, Izmir Democracy University, Karabaglar, Izmir 35140, Turkey

**Keywords:** activities of daily living, late-life depression, older adults, population-based, public health

## Abstract

**Background:** It is a well-established fact that late-life depression represents a significant public health issue, particularly in low- and middle-income countries experiencing rapid demographic aging. Although its clinical and societal impacts are well-recognized, data on the interplay between depressive symptoms and functional status in older populations remain limited for Türkiye. This study aimed to estimate the prevalence of depression among individuals aged 65 years or older, examine its associations with instrumental and basic activities of daily living, and identify key sociodemographic and behavioral correlates. **Methods:** In this study, data obtained from a population-based survey in 2264 clusters by the Turkish Statistical Institute (TUIK) were used, and weighted data were yielded from 6,036,396 adults aged 65 and over. Depression was measured using the Geriatric Depression Scale (GDS), categorizing participants as “not depressed”, “mildly depressed”, or “severely depressed”. Functional status was evaluated using the Lawton–Brody Instrumental Activities of Daily Living (IADL) Scale and the Katz Activities of Daily Living (ADL) Scale. Logistic regression models, adjusted for age and body mass index (BMI), were used to determine the associations of depression with functional impairment and various covariates, including gender, education, marital status, chronic disease, physical activity, smoking, and alcohol use. **Results:** Overall, the prevalence of depression in this cohort was 49.9% [95% CI = 48.7–51%], with 36.0% [95% CI = 34.8–37.0%] classified as mild and 13.9% [95% CI = 13.1–14.7%] as severe depression. IADL and ADL scores were negatively correlated with GDS scores (r = −0.416 and r = −0.321, respectively; *p* < 0.001). In logistic models, lower IADL scores were linked to higher odds of mild (OR = 0.797, 95% CI = [0.796–0.798], *p* < 0.001) and severe depression (OR = 0.689, 95% CI = [0.688–0.690], *p* < 0.001). Being semi-dependent or dependent in ADL further escalated depression risk. Female gender, lower education, single/divorced status, chronic disease, and inactivity also emerged as strong predictors. **Conclusions:** The findings of this study suggest that depression is highly prevalent among older adults in Türkiye, with functional impairment, unfavorable health behaviors, and sociodemographic vulnerabilities heightening risk. Integrating depression screening into geriatric care—alongside interventions to maintain functional independence—may help mitigate the burden of late-life depression in similar contexts.

## 1. Introduction

The world’s population is aging at an unprecedented rate, bringing increased attention to mental health in later life [1,2]. In the International Classification of Diseases, 11th Revision (ICD-11), a depressive episode is defined as at least two weeks of depressed mood or markedly reduced interest present most of the day, nearly every day, accompanied by other symptoms—typically reaching a total of five or more—including hopelessness, diminished energy, impaired concentration, psychomotor or vegetative disturbances, or suicidal ideation, leading to substantial impairment in personal, social, or occupational functioning [3]. Depression is among the most prevalent psychiatric conditions in the elderly, affecting approximately 5.7% of adults over 60 years of age globally [4,5]. This translates to tens of millions of older people with depression, contributing substantially to the global responsibility of the disease—in fact, late-life mental disorders (predominantly depression and anxiety) account for over 10% of all disability in people aged ≥60 [6,7]. Recent analyses further suggest that broader definitions of depressive symptoms yield even higher estimates, with over one-third of the world’s older population experiencing some level of depression [8]. These figures highlight that geriatric depression is a major public health concern worldwide, not a benign or inevitable consequence of aging.

Maintaining functional performance in terms of independence, safety, and quality is equally critical for aging populations. The ability to perform basic activities of daily living (ADL)—such as bathing, dressing, eating, and mobility—is a key determinant of an older person’s autonomy and quality of life [9]. International studies indicate that a considerable proportion of seniors struggle with ADLs: roughly 13% to 40% of people over 70 have at least one ADL limitation, depending on the population and assessment criteria [10]. Losing the capacity to carry out ADLs has profound implications: it leads to increased dependence on caregivers, higher risk of institutionalization (e.g., nursing home placement), greater morbidity and mortality, and reduced quality of life [10,11]. Thus, preserving ADL function is widely recognized as a cornerstone of healthy aging, essential for both individual well-being and alleviating burdens on families and healthcare systems.

Depression and functional disability often co-occur in older individuals, and research suggests that they can mutually reinforce each other. On one hand, depression may exacerbate physical frailty and reduce an individual’s motivation or energy to maintain self-care, thereby accelerating declines in ADL capabilities [12]. On the other hand, the experience of ADL impairments—for example, needing help with everyday self-care tasks—can precipitate or worsen depressive symptoms by eroding self-esteem and increasing social isolation. Longitudinal studies support this bidirectional link between late-life depression and disability [13]. Prior research has shown that baseline depressive symptoms can predict subsequent declines in ADL function, while conversely, baseline ADL disabilities significantly increase the odds of later depression [13]. Therefore, this complex interaction can create a vicious cycle in which mental health and physical function deteriorate in tandem, highlighting the importance of integrated approaches to prevention and care.

Multiple sociodemographic and health-related factors influence both depression and functional ability in older adults [14,15]. Women tend to have higher rates of late-life depression than men, a disparity often attributed to longer lifespans and associated stressors [16]. Increasing age itself, along with the accumulation of chronic diseases (multimorbidity), is strongly linked to greater disability and higher depression risk in the elderly [16]. Furthermore, social factors are critical: being widowed, living alone, or lacking social support is associated with higher depression rates among older adults [13,14,17]. Such individuals often report poorer self-rated health and lower engagement in social activities, which have been tied to worse ADL outcomes [12,18]. Recognizing these interrelated determinants is critical for identifying vulnerable subgroups and informing multifaceted interventions to improve both mental well-being and functional status in aging populations.

However, most epidemiological research on late-life depression and disability comes from high-income Western countries, and population-based data from rapidly aging societies in other contexts remain limited. Turkey represents one such context: the share of Turkey’s population aged 65 and above reached 9.7% (approximately 8.3 million people) in 2021 [19]. In 2024, the elderly population rate was reported as 10.6 by the Turkish Statistical Institute (TUIK) [20]. This rate is predicted to be 16.3% in 2040 and is expected to rise markedly in the coming decades [19]. This rapid demographic shift is accompanied by socio-cultural changes—for example, a transition from extended family living to smaller nuclear households and increasing urbanization—that pose new challenges for elder care and support. In line with this evidence base, the present population-based cross-sectional study was conducted in Turkey to assess the prevalence of depression, evaluate ADL performance, and examine associated sociodemographic and health factors in a community-dwelling elderly population.

## 2. Methods

### 2.1. Study Design and Setting

This study is a population-based cross-sectional survey conducted across all settlements within the Republic of Turkey by TUIK, and secondary data provided by TUIK [20] were used in this study. The final analysis in this research specifically focused on participants aged 65 years and older. Fieldwork took place between 23 October and 18 December 2023 by TUIK. Data were gathered by TUIK using both face-to-face and telephone interviews, facilitated by computer-assisted personal interviewing (CAPI) and computer-assisted telephone interviewing (CATI) methods. The nationwide scope of the survey ensured that the findings capture the diverse socioeconomic and geographical contexts in which older adults reside throughout Turkey.

### 2.2. Study Population and Sampling

The study population consisted of all non-institutionalized individuals residing in Turkey, as recorded in the Address-Based Population Registration System (ADNKS), with at least one household member aged 50 years or older. A multi-stage cluster sampling design was employed: First stage (cluster selection): Clusters (blocks) were identified based on NAD (National Address Database) data linked to the ADNKS. Each cluster contained approximately 100 household addresses with at least one individual aged 50 years or older. These clusters were selected with probability proportional to their size. Second stage (household selection): Within each selected cluster, 10 households were chosen via systematic random sampling. By design, 2264 clusters were initially selected in the first stage, and a total of 22,640 household addresses were targeted. To ensure accurate population-level estimates, survey weights were computed following design weighting, nonresponse adjustment, and iterative calibration to align with estimated national and regional population parameters.

To account for potential survey design effects—namely clustering and unequal selection probabilities—and to ensure that our findings were representative of the non-institutionalized Turkish population, we applied a multi-stage weighting procedure that included design weighting, nonresponse adjustment, and iterative calibration (FERTFAKTOR (INDIVIDUALFACTOR)). By aligning estimates with the national population distribution, this process produced nationally representative point estimates and their corresponding 95% confidence intervals, thus mitigating bias and enhancing the validity of survey results. The weighting variable, “FERTFAKTOR (INDIVIDUALFACTOR)”, was calculated in three distinct stages [20]. In the first stage, base weights were determined for each sample unit as the inverse of the final selection probability, incorporating both cluster- and household-level selection probabilities. These base weights were assigned at the household and individual levels. In the second stage, the base weights were adjusted to compensate for sample attrition due to nonresponse. In the final stage, iterative adjustments were performed using an Integrated Calibration method that utilized estimated population proportions. Through this final adjustment coefficient, the survey estimates were aligned with the projected population (excluding institutionalized individuals) and the total number of households across Turkey.

### 2.3. Ethical Considerations

The study was conducted in full accordance with the ethical principles set forth in the Declaration of Helsinki. All analyses were performed on anonymized microdata supplied by the Turkish Statistical Institute. The data employed in this research were of a secondary nature, drawn from the Turkish Türkiye Elderly Profile Research micro datasets provided by the Turkish Statistical Institute (TÜİK). These datasets are made available to researchers upon request and the payment of a specified fee. Since the data are anonymized, no personally identifiable details are revealed, thereby upholding participant confidentiality. As a result, this study utilized a pre-existing, publicly accessible secondary dataset, and did not necessitate any further consent or ethical approval.

### 2.4. Data Collection Procedures and Data Collection Instrument

Data were collected through a structured questionnaire, administered in two main sections: Household-level data: Using a “Household Questionnaire”, basic demographic and household-level information was obtained, typically from a single respondent speaking on behalf of the household. Individual-level data: A “Personal Questionnaire” was administered to each household member. Interviews were primarily conducted face-to-face using computer-assisted personal interviewing (CAPI). If respondents could not be reached in person, computer-assisted telephone interviewing (CATI) was employed. In cases where older individuals were unable to respond due to health problems or disabilities, a knowledgeable proxy respondent was identified; however, subjective or perception-based questions were omitted in such proxy interviews. To enhance response rates and data quality, an informational letter was sent to households prior to the fieldwork, outlining the survey’s purpose, importance, and potential uses of the data. Data were entered directly into portable computers during interviews, with daily quality checks to identify and resolve missing or inconsistent responses. All data were encrypted and stored securely to maintain confidentiality.

The dependent variable in this study is depression, measured by the Geriatric Depression Scale (GDS). The primary independent variables are daily living activities, evaluated via the Katz Activities of Daily Living (ADL) Scale, and instrumental activities of daily living, assessed by the Lawton–Brody Instrumental Activities of Daily Living (IADL) Scale. In addition, various sociodemographic and health-related factors—including age, gender, educational status, marital status, chronic disease presence, level of physical activity, living alone, tobacco use, body mass index (BMI), and alcohol consumption—were included as independent variables.

Geriatric Depression Scale (GDS): Depression in the Turkey Aging Profile Survey was measured using the GDS. Originally developed by Yesavage et al. (1982) [21] to assess depressive symptoms in older adults, the GDS includes 30 items with “yes/no” response options. Its original Cronbach’s alpha reliability coefficient was 0.94. The Turkish adaptation and validation, conducted by Ertan et al. (1997) [22], yielded a Cronbach’s alpha of 0.92. Each depression-oriented answer receives 1 point, while the other response option is scored as 0, resulting in a total depression score ranging from 0 to 30. Based on total scores, respondents are categorized as “not depressed” (0–9 points), “mildly depressed” (10–19 points), or “severely depressed” (≥20 points).

Lawton–Brody Instrumental Activities of Daily Living (IADL) Scale: In the Turkey Aging Profile Survey, the Lawton–Brody IADL Scale was utilized to determine the degree of independence in instrumental daily living tasks [23,24]. Originally developed by Lawton and Brody (1969) [23], it was adapted into Turkish and validated by Güzel, Üner, Turan, and Uçan (2023) [24]. The scale comprises eight items evaluating skills such as telephone use, shopping, meal preparation, housekeeping, laundry, transportation, medication management, and handling finances. Possible total scores range from 0 to 8, with lower scores indicating higher levels of dependence. The original Cronbach’s alpha coefficient for this Turkish adaptation was 0.85, reflecting satisfactory internal consistency.

Katz Activities of Daily Living (ADL) Scale: To assess basic daily living activities, the Katz ADL Scale was employed [25,26]. Developed by Katz (1963) [25] and adapted to Turkish by Özkan Pehlivanoğlu et al. (2018) [26], this instrument focuses on six fundamental activities: bathing, dressing, toileting, transferring, continence, and feeding. Lower scores on the Katz ADL Scale signify greater difficulty or dependence in performing basic daily tasks. Specifically, total scores categorize respondents as dependent (0–2 points), semi-dependent (3–4 points), or independent (5–6 points). The Turkish version’s Cronbach’s alpha coefficient was reported as 0.91, indicating high reliability.

### 2.5. Statistical Analysis

All analyses were conducted on the weighted data to account for clustering and unequal selection probabilities, thereby ensuring nationally representative estimates. Prior to formal analyses, descriptive statistics were computed to characterize the sample’s sociodemographic, lifestyle, and health-related variables. Continuous variables were evaluated for normality using graphical methods (e.g., histograms, Q-Q plots) and the Kolmogorov–Smirnov test; results were presented as means and standard deviations (SDs). Categorical variables were summarized as weighted frequencies and proportions. To explore bivariate associations among key continuous measures—namely depression score, Lawton–Brody IADL, Katz ADL, age, and BMI—we computed Pearson correlation coefficients. Each correlation coefficient (r) was attended by its 95% confidence intervals (CIs). The magnitude and direction of these correlations were visually represented in a correlation matrix (heatmap), facilitating an initial overview of the interrelationships among variables. Functional status was measured using Lawton IADL and treated as a continuous variable. Katz ADL was categorized as independent, semi-dependent, or dependent for logistic regression. Depressive symptoms were assessed using the GDS, which was further categorized into three groups: not depressed (reference), mild depression, and severe depression. For multivariable models, these categories were dichotomized where appropriate: Model 1: mild depression (vs. not depressed), Model 2: severe depression (vs. not depressed), and Model 3: mild or severe depression (vs. not depressed). Each model was adjusted for age and BMI, given their known associations with both depression and functional status. The dependent variable in each logistic model was the binary outcome contrasting a specific level (or combination) of depression against “Not Depressed”. The multivariable analysis employed a backward stepwise likelihood ratio (LR) method, with odds ratios (ORs) and their corresponding 95% confidence intervals (CIs) calculated for each variable. In the logistic regression models, the intercept (constant) term was included to represent the baseline log odds of the outcome when all predictors are at their reference values; its coefficient (β) and standard error (SE) provide insight into the base likelihood of the dependent variable in the absence of additional covariates. All statistical analyses were conducted using STATA software (v.18, College Station, TX, USA) and the SPSS (v30.0, IBM Corp., Armonk, NY, USA) package program. All tests were two-tailed, and statistical significance in all analyses was quantified at a t *p*-value of < 0.05.

## 3. Results

**Figure 1:**
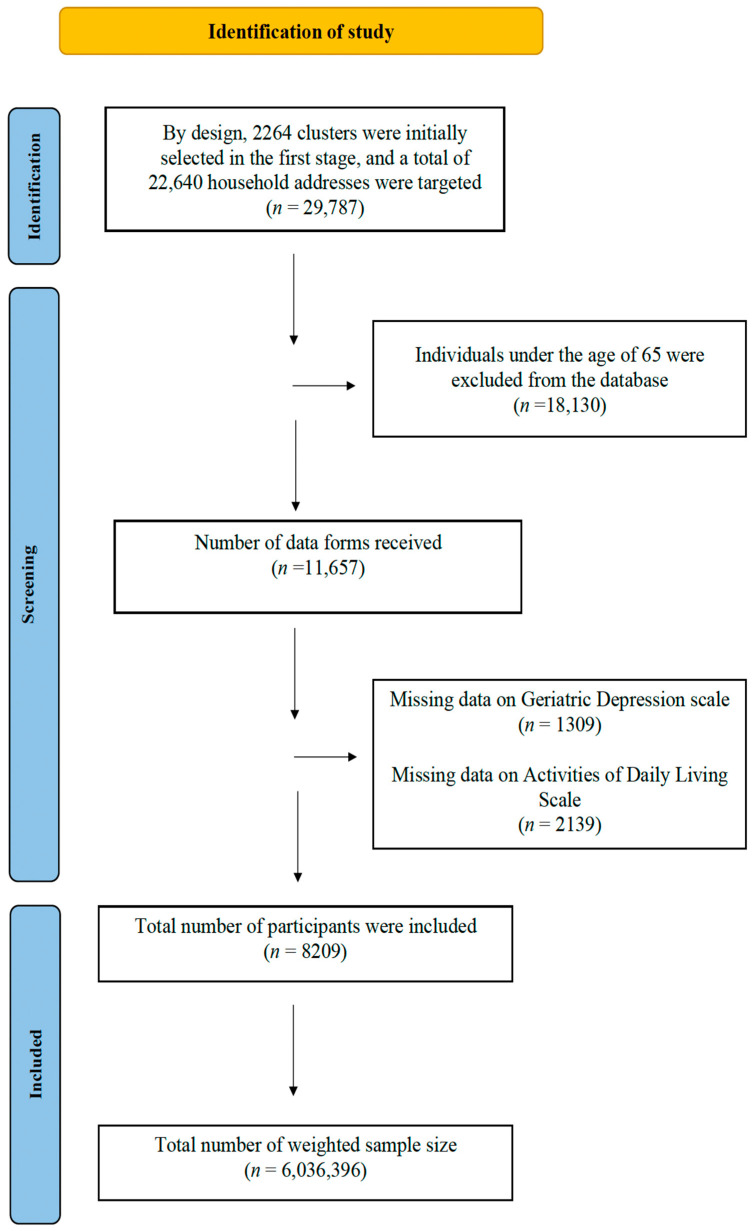
Flowchart illustrating the selection of participants aged 65 years and older in a population-based study.

The step-by-step selection process for participants in this study is illustrated in Figure 1. Initially, 2264 clusters were selected in the first stage, yielding a total of 22,640 household addresses (*n* = 29,787). Of these, 18,130 individuals younger than 65 years were excluded, resulting in 11,657 data forms. Subsequently, participants with missing data on the Geriatric Depression Scale (*n* = 1309) and the Activities of Daily Living Scale (*n* = 2139) were further excluded, leaving a final analytic sample of 8209 individuals. After applying the survey weights, the total weighted sample size was 6,036,396.

Table 1 presents the baseline descriptive and sociodemographic characteristics of the weighted study population (*n* = 6,036,396). The mean age of participants was 73.2 years (SD = 6.5), ranging from 65 to 115 years. Females constituted a larger proportion of the sample (58.5%) compared to males (41.5%), and the majority of older adults did not live alone (77.5%). Likewise, 61.2% were either single or divorced, while 38.8% were married. In terms of education, 92% had attained a high school education or lower, and 8% had completed university or above. Most participants (92.6%) reported at least one chronic disease, reflecting the high burden of chronic conditions in this age group. Regarding health behaviors, 11.8% used tobacco and 12.3% reported alcohol use, whereas 60.9% engaged in predominantly sedentary activities and 71.8% reported no or rare physical activity.

The distribution of GDS, Lawton–Brody IADL, and Katz ADL Scale scores within the weighted study sample is summarized in Table 2. On average, participants scored 10.8 (SD = 6.9) on the GDS, 5.9 (SD = 2.3) on the IADL Scale, and 5.4 (SD = 1.2) on the ADL Scale. The prevalence of at least mild depressive symptoms, based on GDS thresholds, was 49.9%, including 36.0% classified as having mild depression and 13.9% severe depression. Concerning ADL functioning, 89.9% of participants were classified as independent, 4.6% as semi-dependent, and 5.5% as dependent. These findings emphasize a substantial prevalence of depressive symptoms and varying degrees of functional dependence among older adults in the study population.

**Figure 2:**
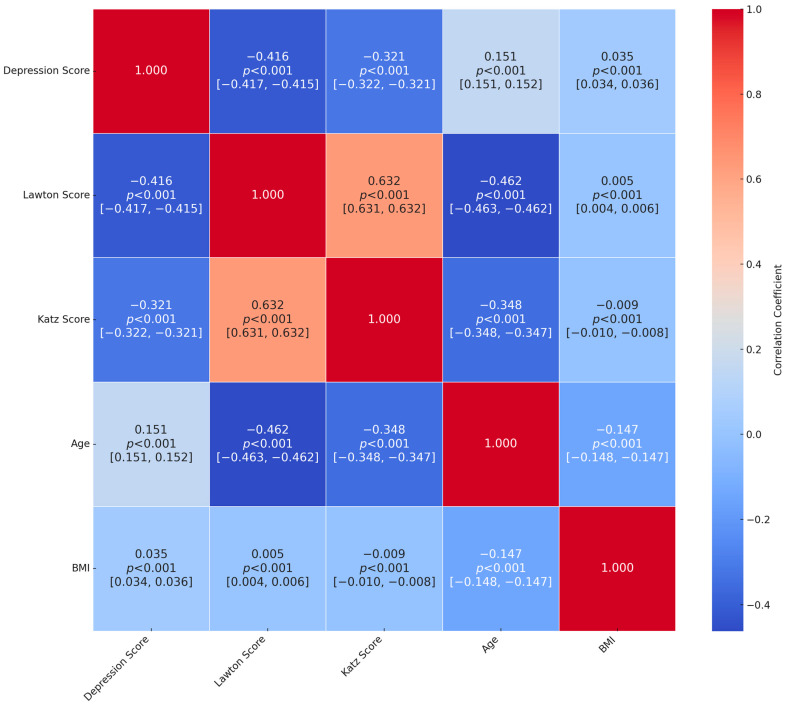
Correlation matrix for depression score, Lawton–Brody Instrumental Activities of Daily Living (IADL) score, Katz Activities of Daily Living (ADL) score, age, and body mass index (BMI), with correlation coefficients and corresponding 95% confidence intervals.

Figure 2 demonstrates the correlation matrix among depression score, instrumental (Lawton–Brody IADL) and basic (Katz ADL) activities of daily living, age, and BMI, along with their 95% CIs. Overall, the depression score showed moderate negative correlations with the Lawton–Brody score (r = −0.416, [95% CI = −0.417, −0.415], *p* < 0.001) and the Katz score (r = −0.321 [95% CI = −0.322, −0.321], *p* < 0.001), indicating that higher functional independence is associated with fewer depressive symptoms. Age had negative associations with both Lawton (r = −0.462 [95% CI = −0.463, −0.462], *p* < 0.001) and Katz scores (r = −0.348 [95% CI = −0.348,−0.347], *p* < 0.001), highlighting the reduction in functional capacity as age increases. Notably, Lawton and Katz scores were strongly positively correlated (r = 0.632 [95% CI = 0.631, 0.632], *p* < 0.001), underscoring the conceptual overlap between instrumental and basic activities of daily living. Finally, BMI exhibited small correlations with the other variables, such as a weak positive association with depression score (r = 0.035 [95% CI = 0.034, 0.036], *p* < 0.001), suggesting that body composition exerts a relatively minimal influence compared to other factors.

The results of three multivariable logistic regression models examining the associations between depression status (mild and/or severe vs. not depressed), activities of daily living (IADL and ADL scores), and various sociodemographic and lifestyle factors in a weighted sample of older adults (*n* = 6,036,396) are presented in Table 3. Model 1 evaluates factors associated with mild depression compared to no depression, controlling for age and BMI. Lower scores on the IADL Scale were significantly linked to higher odds of mild depression (OR = 0.797, 95% CI = [0.796–0.798], *p* < 0.001), indicating that as functional independence decreases, the likelihood of mild depression increases. Similarly, being semi-dependent or dependent in the ADL Scale was associated with elevated odds of mild depression (OR = 1.325, 95% CI = [1.310–1.341] for semi-dependent; OR = 1.467, 95% CI = [1.448–1.485] for dependent), highlighting the role of basic functional impairment. Female gender (OR = 1.483, 95% CI = [1.477–1.490], *p* < 0.001), living single (OR = 1.147, 95% CI = [1.140–1.154], *p* < 0.001), and being single or divorced (OR = 1.130, 95% CI = [1.123–1.136], *p* < 0.001) were also associated with increased odds of mild depression. Moreover, lower educational attainment (high school and below), chronic disease, tobacco use, sedentary lifestyle, and infrequent physical activity further heightened the likelihood of mild depressive symptoms, highlighting the interplay between social, medical, and behavioral factors.

Model 2 focuses on predictors of severe depression relative to no depression, again adjusting for age and BMI. Reduced IADL scores showed an even stronger association with severe depressive symptoms (OR = 0.689, 95% CI = [0.688–0.690], *p* < 0.001) compared to Model 1, suggesting that poorer instrumental functional capacity places individuals at particularly high risk for severe depression. Individuals classified as semi-dependent or dependent in ADL demonstrated markedly greater odds of severe depression (OR = 1.762, 95% CI = [1.739–1.785] and OR = 2.329, 95% CI = [2.297–2.361], respectively), emphasizing the pronounced impact of basic functional impairment on the risk for severe depressive symptoms. Female gender, living single, being single/divorced, and lower educational attainment consistently emerged as risk factors, all with increased odds ratios compared to their associations with mild depression. Additionally, chronic disease, tobacco use, sedentary behavior, and minimal physical activity remained key contributors to severe depression, underscoring the cumulative effect of these demographic and lifestyle variables.

Model 3 combines mild and severe depression into a single outcome category, thereby providing an overview of factors linked to any form of depression. Despite collapsing the two depression categories, lower IADL and ADL scores continued to show strong associations with increased odds of depression (IADL OR = 0.763, 95% CI = [0.762–0.764], *p* < 0.001; ADL-dependent OR = 1.699, 95% CI = [1.679–1.719], *p* < 0.001). Sociodemographic and lifestyle factors—female gender, marital status, educational status, chronic disease, tobacco use, and limited physical activity—were likewise found to elevate the likelihood of experiencing some level of depression. Of note, the odds ratios in Model 3 typically fell between the values reported for mild and severe depression in Models 1 and 2, reinforcing the notion that greater functional impairment and adverse health behaviors correlate with a higher overall risk of depressive symptoms.

## 4. Discussion

In this population-based study of Turkish adults aged 65 and above, we found a remarkably high prevalence of depressive symptoms, affecting nearly half (49.9%) of the elderly population. Specifically, 36.0% screened positive for mild depression and 13.9% for severe depression, indicating that a substantial proportion of community-dwelling seniors experience clinically relevant depressive mood disturbances. These findings underscore depression as a major public health issue in Türkiye’s aging population. Notably, depressive symptom severity was closely linked to functional status: participants with greater impairments in ADL and IADL had significantly higher depression scores, whereas those with intact functionality tended to be free of depressive symptoms. Multivariable logistic models confirmed that poorer functional status and several sociodemographic and lifestyle factors independently increased the odds of depression—for both mild and severe cases—after adjusting for age and BMI. In summary, the main findings reveal that late-life depression is highly prevalent in this Turkish cohort and is inversely associated with functional ability (higher ADL/IADL independence correlating with lower depression), alongside strong associations with female gender, lower education, being unmarried, having chronic diseases, a sedentary lifestyle, current tobacco use, and infrequent physical activity. These results align with our hypotheses and highlight the multifactorial nature of geriatric depression in the community.

A key observation is the inverse correlation between depression and functional status. Elderly individuals with limitations in ADL and IADL were far more likely to exhibit depressive symptoms, suggesting a tight interplay between physical dependence and mental health. Those who were fully dependent on others for basic ADLs had roughly double the odds of depression compared to independent elders, with the odds of severe depression being even higher (e.g., ADL-dependent seniors showed over a two-fold increase in severe depression risk). Conversely, each one-point increase in the IADL score (indicating better capacity in tasks like shopping or housekeeping) was associated with a substantially lower likelihood of depression. These patterns imply that a loss of autonomy and difficulty in performing daily tasks may precipitate or exacerbate depressive symptoms. The direction of causality cannot be ascertained in this cross-sectional design—it is plausible that physical disability leads to social withdrawal, low self-efficacy, and depressed mood, while depression itself can reduce motivation, energy, and cognitive function, thereby worsening one’s ability to manage ADLs. Indeed, evidence from longitudinal studies indicates a bidirectional relationship: physical disability is a well-established risk factor for later-life depression [27], and depression, in turn, can accelerate functional decline. For example, each additional depressive symptom at baseline has been associated with a ~15% increase in the risk of future disability [28]. Our findings reinforce this vicious cycle, highlighting the need for integrated interventions that address both mental well-being and physical functioning in older adults.

In addition to functional impairment, several sociodemographic factors emerged as significant predictors of depression in this population. The female gender was associated with markedly higher odds of depressive symptoms—women had about 1.6 times the odds of overall depression compared to males and nearly twice the odds of severe depression in the adjusted analysis. This gender disparity is consistent with the international literature, which has long documented the female sex as a strong risk factor for late-life depression [27]. Possible explanations include biological susceptibilities, such as hormonal factors and comorbidities, as well as social determinants: in Türkiye, older women (who constituted ~58.5% of the 65+ demographic in our sample) often face cumulative disadvantages such as lower lifetime education and income, widowhood, and caregiving burdens. Many women in this cohort likely outlived their spouses—the lack of a partner’s support and the experience of bereavement can contribute significantly to depression [29]. Being unmarried (including single, divorced, or widowed status) was associated with higher depression prevalence in our study, echoing findings that older adults without a spouse have greater loneliness and approximately a 30–40% higher risk of depression than married peers [29]. Educational level showed an especially pronounced association: individuals with low education (high school or below) had significantly higher odds of depression, and this gap was most extreme for severe depressive symptoms. Those with lower education had over three times the odds of severe depression relative to those with a university education. This steep educational gradient aligns with observations in other countries that low educational attainment is linked to elevated depression risk in old age [30]. Limited education may serve as a proxy for lower socioeconomic status, reduced health literacy, and fewer coping resources, all of which can render elders more vulnerable to mental distress. Additionally, less educated seniors in Türkiye may have worked in physically demanding jobs or have less access to quality healthcare and social support, contributing to both worse physical health and higher depression. Taken together, these demographic patterns suggest that socially disadvantaged groups—particularly older women with less education and those lacking familial support—bear a disproportionate burden of depression, likely reflecting underlying gender roles and health disparities in the aging population.

Unhealthy and sedentary lifestyle factors were also strongly associated with depression in our study, pointing to modifiable risk factors for intervention. Physical inactivity showed one of the largest effects on mental health: older adults who never or rarely engaged in leisure-time physical activity had 1.6 times greater odds of depression overall and nearly double the odds of severe depression compared to those exercising at least once per week. Similarly, those who spent most of their day sitting (sedentary behavior) were significantly more likely to report depressive symptoms than those whose daily activities required at least moderate physical effort. These findings are in line with extensive evidence that regular exercise and staying physically active contribute to better mental health in later life [31]. Even low levels of activity (e.g., walking or light chores) have been shown to reduce depression risk in older adults, likely by improving physiological health, preserving mobility, and providing psychosocial stimulation [31]. Conversely, a sedentary lifestyle can worsen chronic health conditions and isolate individuals socially, thereby increasing the likelihood of depression. Tobacco use was another notable factor. Current smokers had higher rates of depressive symptoms than non-smokers, with an adjusted odds ratio of around 1.1–1.3 for depression. While the effect size was modest, this association is consistent with research linking smoking to depression through multiple pathways. Smoking can contribute to cerebrovascular disease and neurochemical changes that heighten depression risk [32]. Conversely, people with depression may be more prone to smoking as a maladaptive coping mechanism, suggesting a reciprocal relationship. Our findings reinforce the importance of healthy behaviors in maintaining mental well-being. Promoting physical activity, smoking cessation, and other healthy lifestyle changes among seniors could potentially ameliorate depressive symptoms and improve overall quality of life. Encouragingly, these are modifiable factors, so targeted interventions in these areas may yield tangible benefits for geriatric depression prevention and management.

When comparing our results to the existing international literature, we find broad agreement on the risk factors for late-life depression, alongside some differences in prevalence that warrant discussion [33,34]. However, differences in prevalence across countries are notable—for example, rates range from 7% in Italy to 37% in Chile, potentially due to variations in assessment tools, healthcare access, or cultural stigma surrounding mental health [33,35,36] (Field, 2023). The profile of depression correlates identified in this Turkish sample mirrors patterns observed in many other countries. For example, a recent study confirmed that women, individuals with low education, and those with functional disabilities have higher odds of depression in old age across diverse settings [30]. Our evidence linking chronic disease with depression is likewise consistent with prior studies showing that medical comorbidities are major determinants of geriatric depression [37]. The robust association we observed between physical functional impairment and depression echoes findings from both Western and Asian populations, reinforcing that the interplay between disability and depression is a universal phenomenon in aging [28,30]. Likewise, the beneficial role of physical activity and the deleterious impact of a sedentary lifestyle on elder mental health have been documented globally, with numerous studies demonstrating that exercise is associated with lower depression rates among older adults [31]. These consistencies suggest that, despite cultural and environmental differences, the fundamental risk factors and correlates of late-life depression are largely similar across populations.

Our study of depression prevalence appears similar to that reported in many high-income settings and even somewhat similar to the pooled estimates for low- and middle-income countries (LMICs). The overall prevalence of severe depression, 13.9% in Turkish older people, is similar to the 10–20% prevalence range that the World Health Organization has estimated for depressive disorders in the global older population [38]. This study noted that studies from developing regions tend to report higher late-life depression prevalence (around 40% on average) than those from developed countries (~17%), partly due to differences in measurement tools and socioeconomic contexts. Our findings align with this pattern, as Türkiye (an upper-middle-income country) shows a depression burden on the higher end, especially when taking into account mild depression, comparable to reports from some other LMIC contexts. For instance, community studies in South and East Asia have also found very high rates of depressive symptoms among older adults when using screening scales—a rural Chinese sample showed a 53% prevalence of depressive symptoms, very similar to what we observed [30]. These elevated rates in developing settings may reflect unmet mental health needs, greater social stressors, or the inclusion of milder cases that might not be counted in clinical diagnoses. In our study, the use of the GDS as a screening instrument likely captured a broad spectrum of depressive symptoms, including subthreshold cases, thus inflating the prevalence relative to studies that define depression via diagnostic interviews. Cultural factors could also influence reporting—for example, somatic presentations of depression or differing stigma levels might affect how older individuals endorse symptoms on questionnaires. It suggests that late-life depression in Türkiye might be under-recognized and under-treated, pointing to a potential gap in geriatric mental health services. In comparison to Western populations where routine depression screening and treatment for seniors are more established, Turkish older adults may have faced historical barriers to access to mental health care, resulting in a larger reservoir of unmet needs. Further cross-national research would be useful to verify these prevalence differences and explore the cultural and health system factors that contribute to them.

The present findings carry important public health and policy implications for Türkiye and other societies with aging populations [39]. First and foremost, the high prevalence of late-life depression calls for strengthened efforts in the screening, prevention, and treatment of mental health conditions among older adults [39,40]. Health authorities could integrate routine depression screening into primary care visits for seniors, especially in the context of chronic disease management or geriatric assessments, given the strong links we found between depression and physical health status [40,41]. Training primary care physicians and community health workers in geriatric mental health will be crucial so that depressive symptoms are recognized as part of routine elder care [39]. The associations with functional impairment suggest that interdisciplinary care approaches are needed: for example, rehabilitation services and home care programs should monitor clients for mood changes, and conversely, mental health professionals should consider patients’ functional needs. Interventions that maintain or improve functional ability—such as physiotherapy, occupational therapy, and assistive devices for ADL/IADL support—might have ancillary benefits in reducing depression or preventing its onset [39,40]. Likewise, our findings highlight several modifiable risk factors (physical inactivity, smoking, and social isolation due to widowhood or living alone) that policymakers could target through health promotion and social support programs. Community-based exercise programs tailored for seniors, walking groups, and physical activity encouragement in social centers could help break the cycle of sedentary behavior and depression. Tobacco cessation campaigns might be extended to older populations with appropriate messaging, as quitting smoking even in later life can improve overall health and possibly mental well-being. Beyond individual behaviors, strengthening social support for vulnerable elders is essential. For example, widowed or isolated older adults could benefit from organized community groups, senior centers, or volunteer outreach that provide companionship and a sense of purpose, thereby mitigating loneliness and depressive feelings. Culturally, family plays a central role in elderly care in Türkiye; public health strategies can build on this by educating families about the signs of depression and encouraging them to facilitate treatment rather than viewing depression as a normal part of aging or something to be hidden [42]. At the policy level, integrating mental health into aging policies—such as Turkey’s national action plans for older people—will ensure that resources are allocated for geriatric mental health services. In sum, addressing late-life depression requires a comprehensive approach that spans healthcare, community support, and healthy aging initiatives [43]. By tackling the identified risk factors (both medical and social) and improving access to care, policymakers can help improve the quality of life and functional capacity of older adults, ultimately promoting healthy aging in line with global recommendations.

Despite its contributions, this study has several limitations that should be acknowledged when interpreting the results. First, the cross-sectional design precludes any causal inferences—while we observed strong associations, we cannot determine temporality or directionality. Depression and functional impairment likely influence each other over time, and longitudinal studies are needed to disentangle cause and effect. Second, all data on depressive symptoms, functional status, and lifestyle factors were based on self-reports (e.g., questionnaire assessments of mood and ADLs), which introduces potential reporting bias. Elderly respondents may underreport or overreport symptoms due to recall issues or social desirability; for instance, some may be reluctant to admit to depressive feelings due to stigma, whereas others with depression might inadvertently rate their functional abilities worse than objective measures would indicate. The use of the GDS (a screening instrument) is a practical strength for large surveys, but it is not equivalent to a clinical diagnostic evaluation—thus, our “depression” variable includes individuals with elevated depressive symptoms but not necessarily a diagnosed depressive disorder. This could lead to misclassification or an overestimation of prevalence, as milder cases were counted. Similarly, functional ability was measured via standard ADL/IADL scales, which rely on respondents’ or caregivers’ judgment and might not capture nuanced performance ability; subtle cognitive impairments could also affect how these questions were answered. Another limitation is the possibility of residual confounding. We adjusted for major covariates (age and BMI in the models, along with others through stepwise selection), but there may be unmeasured factors influencing both depression and functional status—for example, cognitive decline, pain levels, quality of social support, or personality traits were not directly measured and could bias associations. The dataset was weighted to represent the national elderly population, which is a strength, but we did not explicitly analyze urban–rural differences or regional variations that might exist within Türkiye. Additionally, the study’s context (Turkey’s community-dwelling older adults) may limit generalizability to very different settings; cultural factors and healthcare infrastructure can modulate the patterns of late-life depression, so our findings may not fully apply to countries with distinct socio-cultural environments or to institutionalized elderly (who were not the focus here). Finally, while our sample size was very large, allowing for precise estimates, the cross-sectional nature means that respondents who were too ill or severely cognitively impaired might have been less likely to participate, potentially underestimating associations if the most depressed or frail individuals were missing. Recognizing these limitations, the results should be viewed as indicative of associations rather than proof of causality. Future research that follows older adults over time will be invaluable to confirm these relationships and address the temporal dynamics between depression and declining function. Of note, this study did not investigate the influence of contextual and environmental factors—particularly building accessibility and social support—which prior evidence indicates are critical for mitigating late-life depressive symptoms and for improving aging-in-place outcomes. This omission should be acknowledged in the limitations section, and future research is strongly encouraged to examine these variables in depth. Finally, although the parent survey sampled adults aged 50 years and older, the present analysis was restricted to respondents aged ≥65 years, which may limit the generalizability of our findings to the younger segment of the older-adult population.

## 5. Conclusions

This study provides a comprehensive epidemiological assessment of depression and its associated factors in the older population of Türkiye, revealing a high burden of depressive symptoms linked with functional disability and various modifiable risk factors. Moving forward, longitudinal studies are needed to clarify causality—whether enhancing physical functioning prevents depression or vice versa—and to assess long-term intervention effects. Such research could explore integrated programs combining exercise, social engagement, and counseling aimed at reducing depressive symptoms and preserving functional capacity. Given the multifaceted nature of late-life depression, integrated care appears to be the most promising strategy, bridging geriatric medicine, primary care, rehabilitation, and mental health services to provide coordinated, patient-centered support. In line with the World Health Organization’s framework of integrated care for older people, it should incorporate routine depression screening within elder care services while also focusing on mobility, nutrition, and social connectivity. Ultimately, tackling depression among older adults extends beyond isolated psychiatric treatment, requiring a synergistic approach that promotes functional health, manages chronic diseases, and bolsters social support. By embracing such measures, it can improve the mental well-being of a rapidly aging population, enabling older adults to maintain active, fulfilling lives in the community.

## Figures and Tables

**Table 1:** Baseline descriptive and sociodemographic characteristics of the study group.

Variables	Total (*n* = 6,036,396)
Age, mean ± SD, years	73.2 ± 6.5 (min-max: 65–115)
Gender, *n* (%)	
Male	2,507,653 (41.5)
Female	3,528,743 (58.5)
Live alone or with someone, *n* (%)	
Single	1,360,907 (22.5)
Unsingle	4,675,490 (77.5)
Marital status, *n* (%)	
Married	2,343,353 (38.8)
Single or divorced	3,693,044 (61.2)
Educational status, *n* (%)	
High school and below	5,554,334 (92)
University and above	482,062 (8)
Chronic disease	
No	444,083 (7.4)
Yes	5,592,314 (92.6)
Tobacco use, *n* (%)	
No or quit	5,324,768 (88.2)
Yes	711,629 (11.8)
Alcohol use, *n* (%)	
No	5,294,228 (87.7)
Yes	742,168 (12.3)
Activity during the day, *n* (%)	
Mostly sitting	3,677,483 (60.9)
Requiring moderate or greater force	2,358,913 (39.1)
Physical activity, *n* (%)	
None or rarely	4,334,532 (71.8)
At least once a week or more	2,358,913 (28.2)

The data represent a weighted sample. SD: standard deviation.

**Table 2:** Distribution of Geriatric Depression Scale (GDS), Lawton–Brody Instrumental Activities of Daily Living Scale (IADL), and Katz Activities of Daily Living (ADL) Scale scores of the research group.

Variables	Total (*n* = 6,036,396)
GDS scores, mean ± SD	10.8 ± 6.9 (min–max: 0–30)
IADL scale scores, mean ± SD	5.9 ± 2.3 (min–max: 0–8)
ADL scale scores, mean ± SD	5.4 ± 1.2 (min–max: 0–6)
Status of Geriatric Depression Scale scores, *n* (%)	
Not depressed	3,025,002 (50.1)
Mild depression	2,170,866 (36.0)
Severe depression	840,528 (13.9)
Status of ADL scores, *n* (%)	
Independent	5,425,109 (89.9)
Semi-dependent	280,228 (4.6)
Dependent	331,060 (5.5)

The data represent a weighted sample. GDS: Geriatric Depression Scale; IADL: Lawton–Brody Instrumental Activities of Daily Living Scale; ADL: Katz Activities of Daily Living Scale; SD: standard deviation.

**Table 3:** Multivariable logistic regression analyses of the association between depression, activities of daily living, and associated factors (*n* = 6,036,396).

	Model 1 ^a^	Model 2 ^b^	Model 3 ^c^
Variables	OR [95% CI] *	OR [95% CI] *	OR [95% CI] *
IADL scores	0.797 [0.796–0.798]	0.689 [0.688–0.690]	0.763 [0.762–0.764]
*p*-value	<0.001	<0.001	<0.001
ADL scores			
Independent (reference)	–	–	–
Semi-dependent	1.325 [1.310–1.341]	1.762 [1.739–1.785]	1.455 [1.439–1.470]
Dependent	1.467 [1.448–1.485]	2.329 [2.297–2.361]	1.699 [1.679–1.719]
*p*-value	<0.001	<0.001	<0.001
Gender			
Male (reference)	–	–	–
Female	1.483 [1.477–1.490]	1.966 [1.952–1.980]	1.600 [1.593–1.607]
*p*-value	<0.001	<0.001	<0.001
Live alone or with someone			
Unsingle (reference)	–	–	–
Single	1.147 [1.140–1.154]	1.701 [1.686–1.715]	1.262 [1.255–1.269]
*p*-value	<0.001	<0.001	<0.001
Marital status			
Married (reference)	–	–	–
Single or divorced	1.130 [1.123–1.136]	1.139 [1.129–1.148]	1.136 [1.130–1.142]
*p*-value	<0.001	<0.001	<0.001
Educational status			
University and above (reference)	–	–	–
High school and below	1.515 [1.503–1.526]	3.436 [3.372–3.502]	1.676 [1.664–1.688]
*p*-value	<0.001	<0.001	<0.001
Chronic disease			
No (reference)	–	–	–
Yes	1.383 [1.373–1.394]	1.397 [1.380–1.414]	1.367 [1.358–1.377]
*p*-value	<0.001	<0.001	<0.001
Tobacco use			
No or quit (reference)	–	–	–
Yes	1.087 [1.081–1.094]	1.280 [1.268–1.292]	1.135 [1.128–1.141]
*p*-value	<0.001	<0.001	<0.001
Alcohol use			
No (reference)	–	–	–
Yes	1.039 [1.033–1.046]	0.971 [0.960–0.981]	1.037 [1.030–1.043]
*p*-value	<0.001	<0.001	0.029
Activity during the day			
Requiring moderate or greater force (reference)	–	–	–
Mostly sitting	1.381 [1.375–1.387]	1.741 [1.729–1.753]	1.449 [1.443–1.454]
*p*-value	<0.001	<0.001	<0.001
Physical activity			
At least once a week or more (reference)	–	–	–
None or rarely	1.570 [1.563–1.577]	1.992 [1.976–2.008]	1.635 [1.628–1.642]
*p*-value	<0.001	<0.001	<0.001
	Constant β = 1.111 (SE = 0.017) *p*-value < 0.001	Constant β = 1.718 (SE = 0.026) *p*-value < 0.001	Constant β = 1.959 (SE = 0.016) *p*-value < 0.001

The data represent a weighted sample. ^a^ Multivariable logistic regression analysis of the association between mild depression (vs. not depressed), activities of daily living, and associated factors (Model 1 was adjusted by age and BMI). ^b^ Multivariable logistic regression analysis of the association between severe depression (vs. not depressed), activities of daily living, and associated factors (Model 2 was adjusted by age and BMI). ^c^ Multivariable logistic regression analysis of the association between mild and severe depression (vs. not depressed), activities of daily living, and associated factors (Model 3 was adjusted by age and BMI). OR: odds ratio; CI: confidence interval; SE: standard error; OR: odds ratio; CI: confidence interval. * Multivariable logistic regression analysis calculated by using the backward stepwise likelihood ratio (LR) method. IADL: Lawton–Brody Instrumental Activities of Daily Living Scale. ADL: Katz Activities of Daily Living Scale.

## Data Availability

The datasets used and/or analyzed in this study are available upon reasonable request from the Turkish Statistical Institute (TÜİK).
